# Sources of Fine Particulate Matter and Risk of Preterm Birth in Connecticut, 2000–2006: A Longitudinal Study

**DOI:** 10.1289/ehp.1307741

**Published:** 2014-05-23

**Authors:** Gavin Pereira, Michelle L. Bell, Hyung Joo Lee, Petros Koutrakis, Kathleen Belanger

**Affiliations:** 1 Center for Perinatal Pediatric and Environmental Epidemiology, School of Medicine, Yale University, New Haven, Connecticut, USA; 2 School of Forestry and Environmental Studies, Yale University, New Haven, Connecticut, USA; 3 Department of Environmental Health, Harvard School of Public Health, Boston, Massachusetts, USA

## Abstract

Background: Previous studies have examined fine particulate matter (≤ 2.5 μm; PM_2.5_) and preterm birth, but there is a dearth of longitudinal studies on this topic and a paucity of studies that have investigated specific sources of this exposure.

Objectives: Our aim was to assess whether anthropogenic sources are associated with risk of preterm birth, comparing successive pregnancies to the same woman.

Methods: Birth certificates were used to select women who had vaginal singleton live births at least twice in Connecticut during 2000–2006 (*n* = 23,123 women, *n* = 48,208 births). We procured 4,085 daily samples of PM_2.5_ on Teflon filters from the Connecticut Department of Environmental Protection for six cities in Connecticut. Filters were analyzed for chemical composition, and Positive Matrix Factorization was used to determine contributions of PM_2.5_ sources. Risk estimates were calculated with conditional logistic regression, matching pregnancies to the same women.

Results: Odds ratios of preterm birth per interquartile range increase in whole pregnancy exposure to dust, motor vehicle emissions, oil combustion, and regional sulfur PM_2.5_ sources were 1.01 (95% CI: 0.93, 1.09), 1.01 (95% CI: 0.92, 1.10), 1.00 (95% CI: 0.89, 1.12), and 1.09 (95% CI: 0.97, 1.22), respectively.

Conclusion: This was the first study of PM_2.5_ sources and preterm birth, and the first matched analysis, that better addresses individual-level confounding potentially inherent in all past studies. There was insufficient evidence to suggest that sources were statistically significantly associated with preterm birth. However, elevated central estimates and previously observed associations with mass concentration motivate the need for further research. Future studies would benefit from high source exposure settings and longitudinal study designs, such as that adopted in this study.

Citation: Pereira G, Bell ML, Lee HJ, Koutrakis P, Belanger K. 2014. Sources of fine particulate matter and risk of preterm birth in Connecticut, 2000–2006: a longitudinal study. Environ Health Perspect 122:1117–1122; http://dx.doi.org/10.1289/ehp.1307741

## Introduction

Among almost all high- and middle-income countries, preterm birth is the leading cause of child mortality (Goldenberg et al. 2008; Liu et al. 2012). The United States is of particular interest because it is a high-income country yet has a high preterm birth rate (12%), accounting for more than half a million cases in 2010 (Blencowe et al. 2012). It has been suggested that exposure to ambient air pollution might explain a fraction of adverse birth outcomes such as preterm birth (Pereira et al. 2010; Shah and Balkhair 2011; Stieb et al. 2012). Notably, elevated mass concentrations of fine particulate matter (PM_2.5_), defined as airborne particles of aerodynamic diameter ≤ 2.5 μm, have been associated with preterm birth in various studies (Brauer et al. 2008; Chang et al. 2012; Huynh et al. 2006; Ritz et al. 2007). It is unclear whether such associations reflect true causal effects as the women with the highest exposure have other sociodemographic risk factors (Bell and Ebisu 2012; Goldenberg et al. 2008). A more causal approach to the problem can be achieved by comparing pregnancies to the same woman, which inherently accounts for time-invariant confounders at the individual level. We previously observed adverse associations between preterm birth and PM_2.5_ total mass in Connecticut using a longitudinal design (Pereira et al. 2014). In that study, pregnancies with elevated PM_2.5_ total mass exposure were more likely to result in preterm birth than other pregnancies to the same women at lower levels of exposure. However, PM_2.5_ exhibits large spatiotemporal variation in its chemical composition, which cannot be described using total mass measurements alone (Bell et al. 2007). Associations between preterm birth and PM_2.5_ might reflect effects of the chemical signatures of PM_2.5_ that identify prominent sources of exposure. Source-specific studies have great potential to elucidate the pathways by which fine particulate matter might lead to preterm birth. Because PM is a chemically nonspecific pollutant (with standards currently based on mass concentration), elucidating the effects of sources might also prove valuable from a regulatory perspective.

The aim of this study was to identify major sources of PM_2.5_ in Connecticut and assess the hypothesis that exposures to the anthropogenic sources are associated with elevated risk of preterm birth.

## Methods

*Study design and population*. This was a longitudinal study of a retrospective cohort—retrospective because records were obtained after the women delivered their children, and longitudinal by repeated pregnancies to the same women over time. We investigated women’s exposure to sources of ambient PM_2.5_ and preterm birth across successive pregnancies in Connecticut, 2000–2006. From a population of 271,204 singleton live births without congenital anomaly we sequentially excluded 157 (0.06%) birth records with missing gestational age; 1,846 (0.86%) records that could not be geocoded; 1,505 (0.56%) birth records to women who resided farther than 40 km from a monitoring station for PM_2.5_ mass concentration; 1,443 (0.54%) records with missing parity; and 72,362 (27.18%) births by cesarean delivery. The remaining reference population consisted of birth records for 193,891 neonates to 152,934 women.

For the longitudinal analyses, this reference population was further sequentially restricted to records where at least 75% of the weekly mass concentrations were available in each trimester and whole pregnancy (24,732 births excluded), women who lived within 40 km of a PM_2.5_ monitor for which PM_2.5_ chemical components were measured (21,843 births excluded), and, finally, women who gave birth at least twice during the study period (99,108 births excluded). This resulted in a final study population of 48,208 neonates born to 23,123 women.

*Data sources and variables*. Birth records were obtained from the Connecticut Department of Health (Hartford, CT) for all registered births in Connecticut from 1 January 2000 to 31 December 2006. Each observation contained variables for the residential location at birth; pregnancy-related risk factors (e.g., average number of cigarettes smoked per day during pregnancy, birth order); and sociodemographic risk factors (e.g., race/ethnicity, maternal age).

*Outcome assessment*. Preterm birth was defined as birth before 37 weeks completed gestation. Period of gestation was obtained from the birth certificate record. This was the clinical best estimate of gestational age, based on ultrasound or last menstrual period if ultrasound was not available. More than 90% of all births in Connecticut have a dating ultrasound. First and second trimesters were defined as weeks 1–13 and 14–26, respectively. Third trimester was defined as commencing week 27 and ending at the end of week 36 or birth, whichever was earlier.

*Sampling and chemical analysis*. PM_2.5_ samples, collected on Teflon filters, were obtained from the Connecticut Department of Energy & Environmental Protection (Hartford, CT) for six sites (in Bridgeport, Danbury, Hartford, New Haven, Norwalk, and Waterbury) for the period 2000–2006. The 24-hr integrated PM_2.5_ samples were taken every third day in Bridgeport (692 samples), Danbury (667 samples), Hartford (694 samples), New Haven (686 samples), Norwalk (683 samples), and Waterbury (663 samples). These filters were originally used by the Connecticut Department of Energy & Environmental Protection to measure PM_2.5_ total mass according to federal U.S. Environmental Protection Agency (EPA) sampling protocol (U.S. EPA 2013). The filter samples were analyzed for levels of PM_2.5_ elements using X-ray fluorescence (XRF) for 51 elements (Watson et al. 1999) and optical reflectance for black carbon (BC) concentrations (Cyrys et al. 2003). XRF was performed at the Desert Research Institute, Reno, Nevada, and optical reflectance at the Environmental Chemistry Laboratory, Environmental Health Department of the Harvard School of Public Health. The minimum detection limit (MDL) for the 51 elements was defined as three times the analytical uncertainty. The MDL for BC was set as three times the standard deviation of the values from the optical reflectance analyses of field blanks (Gent et al. 2009). To ensure comparability of source apportionment results to a previously published study, the same criteria for selection of elements for source apportionment was applied (Lee et al. 2011). Briefly, elements were excluded from the source apportionment modeling if > 90% of the samples had values below the MDL unless they were important tracer elements for potential source types. However, tracer elements were not included if all the samples were below the MDL. Samples with values above the 99th percentile of values observed during the study period were excluded as they typically represent atypical sources, such as fireworks or forest fires.

*Source apportionment*. Source apportionment was conducted separately for each monitoring site. Source apportionment was conducted using Positive Matrix Factorization (PMF) with PMF 3.0 developed by the U.S. EPA (2008) using a similar approach to that performed for an earlier period in Connecticut (Lee et al. 2011). Briefly, PMF factorizes a matrix, *X* (*n* samples by *m* species), of observed species concentrations. The result is a representation of *X* as the product of a matrix, *G* (*n* samples by *p* sources), of source contributions for each sample and a matrix, *F* (*p* sources by *m* species), of species profiles for each source plus a matrix of residuals. The matrix of residuals, *E* (*n* samples by *m* species), contains the differences between the observed and modeled concentrations of each species for each sample.

*X* = *GF* + *E.* [1]

Elements of *G* and *F* are constrained to be non-negative and minimize the sum of the squared residuals scaled by their standard deviation (uncertainty), *Q*:

*Q* = Σ__∀__*_ij_* [(*X* – *GF*)*_ij_*/Σ*_ij_*]^2^, [2]

where *i* = 1, 2,…, *n* (samples) and *j* = 1, 2,…, *m* (species).

The PMF solution was rotated, with FPEAK parameters within the interval (–0.4, 0) (Paatero et al. 2005). The result of the PMF is the estimated amount of mass attributable to each source for each sample (i.e., source contributions, in micrograms per cubic meter), and the estimated profiles describing how each species mass is distributed over the sources (i.e., source profiles, in percentage). Tracer elements used to identify motor vehicle emissions were BC, zinc (Zn), lead (Pb), copper (Cu), and bromine (Br). Tracer elements for dust were silicon (Si), iron (Fe), barium (Ba), titanium (Ti), manganese (Mn), and alkaline earth metals of dust from road coverings aluminum (Al), calcium (Ca), and potassium (K). Tracer elements for oil combustion were vanadium (V) and nickel (Ni). For sea salt, tracer elements were sodium (Na) and chlorine (Cl). Sulfur (S) was the major tracer element for regional sulfur factor.

*Exposure assessment*. Weekly mean concentrations of the identified PM_2.5_ source contributions (obtained at a frequency of 2–3 values per week) were calculated as 7-day averages of the daily source contributions for each monitor over the 2000–2006 period. Mean exposures were computed for each week of gestation and then used to compute exposure for each trimester and whole pregnancy. Only exposure estimates to week 36 were included in third trimester and whole pregnancy. Given previously observed associations between preterm birth and PM_2.5_ mass concentration, we assigned exposure estimates to each woman based on the closest monitor within 40 km of the women’s residence at time of birth (Pereira et al. 2014).

*Statistical methods and analyses*. Pregnancies were matched by mother and statistical associations investigated using conditional logistic regression using SAS version 9.3 (SAS Institute Inc., Cary, NC). Separate models were fitted for each trimester and whole pregnancy exposure. Adjustment was made for average number of cigarettes smoked per day (none, 1–9, 10–20, > 20 cigarettes/day), maternal age (< 20, 20–24, 25–29, 30–34, 35–39, ≥ 40 years), and parity (0, 1, 2, ≥ 3 children) for each pregnancy. Adjustment was made for these factors because of their potential to change considerably between pregnancies, and they are strong independent risk factors for preterm birth (Goldenberg et al. 2008; Muglia and Katz 2010). Individual-level education was not included for adjustment as there was negligible change between pregnancies.

*Sensitivity analyses*. We conducted a range of analyses to assess the sensitivity of the observed effect estimates to *a*) choice of the 40-km buffer distance, *b*) smoking status, *c*) certainty of the LMP (last menstrual period) date, *d*) changing residential address between pregnancies, *e*) changing residential address within pregnancies, *f*) exclusion of births by cesarean section, *g*) unmeasured temporal confounders, *h*) adjustment for additional socioeconomic variables (see respective sections in Supplemental Material, Figures S1–S8).

Sensitivity of analyses to buffer distance was investigated by repeating analyses after restriction to women living within 10 km, 20 km, and 30 km from a monitor. Smoking status during pregnancy was ascertained with a binary variable (yes/no). Certainty of LMP date was also ascertained with a binary variable (certain/uncertain). We identified women who changed address between pregnancies using address recorded at delivery, and those who changed address during pregnancy with a variable also recorded at delivery on the number of months the women has resided at the address. We calculated a propensity score for preterm birth conditional on a GAM spline of conception date with an unmatched logistic regression model using the reference population, where the smoothing parameters were selected by generalized cross-validation. This spline term was then used as an adjustment term in the longitudinal analysis to account for potential unmeasured temporal confounders. Sensitivity to additional socioeconomic adjustment was investigated with marital status at the individual level and percent unemployed and median household income at the census-tract level.

*Approvals*. Institutional review board approvals for this study were obtained from the Yale Human Investigation Committee and the Connecticut Department of Health.

## Results

*Preterm birth rate and characteristics of the longitudinal study population*. The preterm birth rate was 7.45% for the original source population before exclusions, 6.40% for the reference population, and 5.36% for the longitudinal study population (see Supplemental Material, Table S1). The longitudinal study design resulted in few women ≥ 40 years of age (*n* = 145, 0.63%) and more women < 20 years of age (*n* = 2,434; 10.53%). Fewer women had < 12 years of education (*n* = 3,290; 14.23%). Most women were married (*n* = 16,138; 69.79%), white (*n* = 15,469; 66.90%), nonsmokers (*n* = 21,802; 94.29%), and had no other children (*n* = 14,741; 63.75%). A small proportion of women delivered only preterm neonates (*n* = 283; 1.22%). There were 1,966 (8.50%) women who delivered both preterm and term neonates.

*Identified sources of PM_2.5_*_._ The proportion of PM_2.5_ component observations below the MDL varied by site (see Supplemental Material, Table S2). The proportion of variation (*R*^2^) in observed PM_2.5_ explained by the PMF solution was 86% in Bridgeport, 92% in Danbury, 88% in Hartford, 74% in New Haven, 92% in Norwalk, and 92% in Waterbury. The source apportionment resulted in detection of five sources (motor vehicle emissions, regional sulfur, oil combustion, dust, and sea salt) in Bridgeport, Danbury, Hartford, and New Haven.

*Spatial variation in sources of PM_2.5_*_._ In general, the factor profiles, which reflect the composition of emissions in each source, (Table 1; see also Supplemental Material, Tables S3–S8) were similar across sites, as previously observed for an earlier period (Lee et al. 2011). However, Ba and Cu had lower contributions to the motor vehicle source and larger contribution to the dust source in New Haven compared with the other sites. This might indicate a relatively larger contribution of road dust particulates to the dust source in New Haven. The source profiles for the oil combustion source were similar among the sites. The sites had similar profiles for the regional sulfur source, although regional sulfur profile for New Haven had relatively greater contributions from Br, Cu, and K. New Haven also had relatively more regional sulfur, possibly due to the influence of local sources near the monitoring site, such as the oil- and natural gas–fired power plant and seaport activity. The distribution of species for the sea salt factor was similar among Bridgeport, Hartford, and New Haven. For Danbury, this factor not only contained tracer elements for sea salt but also included Zn, K, Ni, Pb, and Cu and was therefore not classified as a single source. The species profiles for sources in Norwalk and Waterbury slightly differed from profiles for the other sites because fewer components were used in the source apportionment. The sea salt factor was not identified in Norwalk and Waterbury. Although Norwalk is coastal, the sea salt factor was not identified, possibly because a tracer element (Na) did not meet the inclusion criteria for this site. Based on the contributions of Cl, another tracer for sea salt, the sea salt factor was likely included in the dust source in Norwalk and Waterbury.

**Table 1:** Site-specific concentrations [μg/m^3^ (%)] of PM_2.5_ attributed to each source.

Site	Sea salt	Oil combustion	Motor vehicle emissions	Dust	Regional sulfur	Total
Bridgeport	0.26 (2.21)	1.53 (13.15)	3.71 (31.93)	1.67 (14.43)	4.44 (38.28)	11.61
Danbury	1.17 (10.42)^*a*^	0.75 (6.65)	3.36 (29.82)	1.12 (9.91)	4.87 (43.20)	11.27
Hartford	0.14 (1.39)	1.21 (12.22)	3.38 (34.17)	1.40 (14.11)	3.77 (38.11)	9.90
New Haven	0.45 (3.60)	0.38 (2.98)	3.85 (30.49)	1.15 (9.13)	6.79 (53.80)	12.62
Norwalk		1.69 (14.43)	3.45 (29.44)	2.06 (17.64)	4.51 (38.50)	11.71
Waterbury		2.54 (20.72)	3.57 (29.18)	1.75 (14.25)	4.39 (35.85)	12.25
^***a***^Sea salt plus other nonidentified sources.						

**Figure 1:**
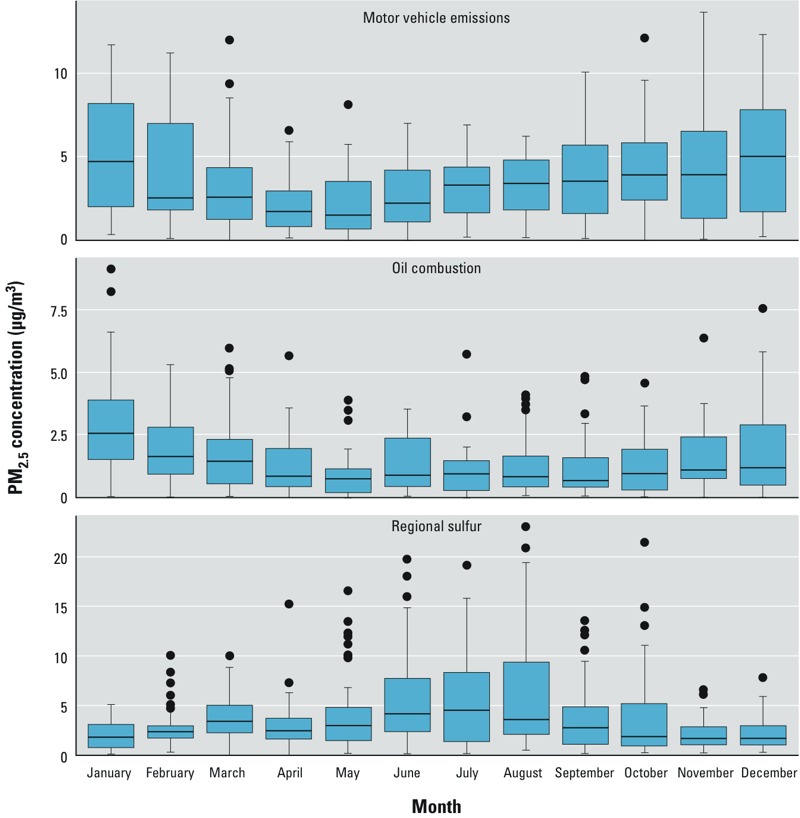
PM_2.5_ concentrations attributable to motor vehicle emissions, oil combustion, and regional sulfur, by calendar month in Hartford, CT. Boxes extend from the 25th to the 75th percentile, horizontal bars represent the median, whiskers extend 1.5 times the length of the IQR above and below the 75th and 25th percentiles, respectively, and outliers are represented as points. The numbers of samples in each month were as follows: January, 37; February, 41; March, 48; April, 49; May, 50; June, 43; July, 40; August, 50; September, 51; October, 47; November, 38; December, 39.

*Temporal variation in anthropogenic sources of PM_2.5_*_._ Regional sulfur, motor vehicle emissions, and oil combustion exhibited the most temporal variation among sources. Regional sulfur peaked in the warm season (June–August), whereas both motor vehicle emissions and oil combustion peaked in the cold season (December–February) (Figure 1, Hartford, CT). Reasons for such temporal variation have been described previously (Lee et al. 2011). Briefly, photochemical activity is greater in the warm season, when more sulfur dioxide is oxidized to sulfate. Motor vehicle emissions peak in the cold season due to both shallower mixing depths and more stable atmospheric conditions. Higher oil combustion levels in the cold season might be explained by space heating with oil boilers, because oil-fired power plant emissions remain relatively constant throughout the year in the region.

*Exposure to anthropogenic sources of PM_2.5_*_._ Throughout pregnancy, women were exposed to higher median levels of regional sulfur (4.26 μg/m^3^) than of motor vehicle emissions (3.52 μg/m^3^), dust (1.49 μg/m^3^), and oil combustion (1.29 μg/m^3^) (Table 2). For each woman, the range in the source exposure was calculated as the pregnancy with highest whole pregnancy exposure minus the pregnancy with the lowest exposure. The mean (± SD) ranges in whole pregnancy exposure to motor vehicle emissions, oil combustion, dust, and regional sulfur were 1.29 ± 0.92 μg/m^3^, 0.65 ± 0.59 μg/m^3^, 0.47 ± 0.39 μg/m^3^, and 1.11 ± 0.87 μg/m^3^, respectively. The mean range in whole pregnancy PM_2.5_ mass concentration was 1.24 ± 0.98 μg/m^3^.

**Table 2:** Distribution of exposure to sources of PM_2.5_ (μg/m^3^) by trimester of exposure.

Exposure	1st trimester			2nd trimester			3rd trimester			Whole pregnancy		
	25th percentile	50th percentile	75th percentile	25th percentile	50th percentile	75th percentile	25th percentile	50th percentile	75th percentile	25th percentile	50th percentile	75th percentile
Oil combustion	0.63	1.15	1.86	0.61	1.16	1.89	0.53	1.07	1.87	0.77	1.29	1.88
Motor vehicle emissions	2.57	3.54	4.52	2.54	3.46	4.40	2.44	3.41	4.35	2.67	3.52	4.11
Dust	1.12	1.42	1.79	1.16	1.44	1.80	1.16	1.45	1.82	1.23	1.49	1.73
Regional sulfur	2.91	4.12	5.78	2.87	4.06	5.69	2.77	3.97	5.76	3.62	4.26	5.28

*Association between anthropogenic sources of PM_2.5_ and preterm birth*. Among all women, the odds ratios (ORs) for preterm birth for IQR increases in whole pregnancy exposure to dust, motor vehicle emissions, oil combustion, and regional sulfur were 1.01 (95% CI: 0.93, 1.09), 1.01 (95% CI: 0.92, 1.10), 1.00 (95% CI: 0.89, 1.12), and 1.09 (95% CI: 0.97, 1.22), respectively (Table 3). Associations for whole pregnancy exposure to dust and motor vehicle emissions were similar for white, Hispanic, and black women. However, the point estimate for an IQR increase in exposure to regional sulfur was higher for Hispanic (OR = 1.28; 95% CI: 0.97, 1.68) and black women (OR = 1.35; 95% CI: 0.97, 1.87) than that for white women, although not statistically significant or statistically different. Similarly, the point estimate for an IQR increase in exposure to oil combustion was higher among black women (OR = 1.43; 95% CI: 0.99, 2.07).

**Table 3:** Adjusted ORs (95% CIs) of preterm birth for IQR increases in anthropogenic sources of PM_2.5_ in Connecticut, 2000–2006.

Group and exposure	1st trimester^*a*^	2nd trimester^*a*^	3rd trimester^*a*^	Whole pregnancy^*a*^
All women^*b*^
Dust	1.03 (0.98, 1.08)	0.98 (0.93, 1.03)	0.99 (0.94, 1.04)	1.01 (0.93, 1.09)
Motor vehicle emissions	1.01 (0.95, 1.07)	0.98 (0.92, 1.04)	1.03 (0.97, 1.09)	1.01 (0.92, 1.10)
Oil combustion	1.03 (0.98, 1.09)	0.96 (0.91, 1.01)	0.99 (0.94, 1.04)	1.00 (0.89, 1.12)
Regional sulfur	1.06 (1.00, 1.12)	0.98 (0.92, 1.03)	1.03 (0.98, 1.09)	1.09 (0.97, 1.22)
White women^*c*^
Dust	1.04 (0.98, 1.10)	0.99 (0.92, 1.06)	1.00 (0.94, 1.07)	1.02 (0.92, 1.13)
Motor vehicle emissions	1.03 (0.96, 1.11)	1.00 (0.93, 1.09)	1.05 (0.97, 1.13)	1.05 (0.93, 1.18)
Oil combustion	1.01 (0.95, 1.08)	0.98 (0.92, 1.05)	0.99 (0.93, 1.06)	0.96 (0.83, 1.12)
Regional sulfur	1.01 (0.94, 1.10)	0.93 (0.86, 1.01)	1.01 (0.94, 1.09)	0.97 (0.83, 1.13)
Hispanic women^*d*^
Dust	1.08 (0.96, 1.22)	0.90 (0.81, 1.00)	0.99 (0.88, 1.12)	1.04 (0.86, 1.24)
Motor vehicle emissions	1.00 (0.87, 1.14)	0.93 (0.80, 1.07)	1.07 (0.93, 1.23)	0.99 (0.80, 1.22)
Oil combustion	1.12 (0.97, 1.30)	0.81 (0.70, 0.93)*	0.98 (0.87, 1.09)	0.95 (0.72, 1.27)
Regional sulfur	1.14 (0.99, 1.31)	0.98 (0.86, 1.12)	1.10 (0.97, 1.25)	1.28 (0.97, 1.68)
Black women^*e*^
Dust	1.01 (0.87, 1.17)	1.03 (0.90, 1.19)	0.91 (0.78, 1.07)	0.99 (0.80, 1.21)
Motor vehicle emissions	0.99 (0.84, 1.16)	0.89 (0.76, 1.04)	0.91 (0.77, 1.07)	0.88 (0.70, 1.10)
Oil combustion	1.14 (0.95, 1.38)	1.02 (0.88, 1.19)	1.03 (0.84, 1.26)	1.43 (0.99, 2.07)
Regional sulfur	1.02 (0.87, 1.20)	1.08 (0.93, 1.26)	1.09 (0.95, 1.26)	1.35 (0.97, 1.87)
ORs compare each woman’s preterm pregnancies to her term pregnancies. ^***a***^Adjusted for parity, maternal age, and tobacco smoking during pregnancy. ^***b***^All women: 48,208 births, 23,123 women, 8.50% of women with both preterm and term births during the study period. ^***c***^White women: 32,346 births, 15,828 women, 7.01% of women with both preterm and term births during the study period. ^***d***^Hispanic women: 8,338 births, 4,155 women, 9.65% of women with both preterm and term births during the study period. ^***e***^Black women: 4,860 births, 2,413 women, 11.94% of women with both preterm and term births during the study period. *p > 0.05, chi-square two-sided tests, except for the OR for second-trimester exposure to oil combustion among Hispanic women.				

Trimester exposures were autocorrelated, but adjustment of trimester effects for exposure in other trimesters minimally influenced results and thus were not considered further. In general there was relatively more evidence for adverse associations for first- and third-trimester exposures than for second-trimester exposure. The ORs for an interquartile range (IQR) increase in first- and third-trimester exposure to regional sulfur were 1.06 (95% CI: 1.00, 1.12) and 1.03 (95% CI: 0.98, 1.09), respectively, with some suggestion of stronger associations among Hispanic women. For exposure to oil combustion, adverse associations were strongest for elevated exposure in first trimester, more prominently among Hispanic (OR = 1.12; 95% CI: 0.97, 1.30) and black women (OR = 1.14; 95% CI: 0.95, 1.38). Among Hispanic women, associations in the opposite direction (OR < 1) were observed for IQR increases in second-trimester exposure to oil combustion (OR = 0.81; 95% CI: 0.70, 0.93) and dust (OR = 0.90; 95% CI: 0.81, 1.00).

*Sensitivity analyses*. The original effect estimates were generally robust to choice of the 40-km buffer distance (see Supplemental Material, Figure S1), the inclusion of smokers in the study population (see Supplemental Material, Figure S2), accuracy of the LMP date (see Supplemental Material, Figure S3), change of residential address either between (see Supplemental Material, Figure S4) or within (see Supplemental Material, Figure S5) pregnancies, adjustment for unmeasured temporal confounders (see Supplemental Material, Figure S7), and adjustment for additional socioeconomic variables (see Supplemental Material, Figure S8). We excluded all cesarean sections from original analyses because final gestational length for cesarean sections without labor is determined before delivery, and Connecticut birth certificates were not suitable for separately identifying cesarean sections that occurred with labor and those that occurred without labor. It is plausible that a fraction of morbidities antecedent of cesarean section were caused by exposure to PM_2.5_ sources, in which case births by cesarean section would not be excluded. However, exposure would be misclassified for all of these individuals in our study because we did not have information on the time of onset/diagnosis of the antecedent during pregnancy. Nonetheless, the original results were robust to the exclusion of births by cesarean section (see Supplemental Material, Figure S6).

## Discussion

*Summary of results*. By matching pregnancies to the same woman, we investigated the hypothesis that women are more likely to deliver preterm when exposure to anthropogenic sources of PM_2.5_ is elevated. This was achieved by longitudinal follow-up of outcomes among > 23,000 women over a 7-year period. This approach lowers the considerable potential for confounding by sociodemographic factors, a limitation of previous studies that relied on between-women comparisons. We also procured > 4,000 daily samples of PM_2.5_ from the Connecticut Department of Energy & Environmental Protection for six cities in Connecticut, which significantly improved both the temporal resolution as well as the geographic relevance of the identified sources of exposure.

The results demonstrated that the greatest contributions to exposure were attributed to regional sulfur and motor vehicle emissions. Concentrations of these sources were more than double the concentrations of sea salt, oil combustion, and dust. The results also showed temporal variation in sources, with peak regional sulfur in the warm season (June–August). Motor vehicle emissions and oil combustion peaked in the cold season (December–February). There was insufficient evidence to conclude that fine particulate levels of dust, motor vehicle emissions, oil combustion, and regional sulfur were associated with risk of preterm birth. Although central estimates for three of the four sources were positive (and the fourth null), they were not statistically significant. Adjusted ORs per IQR increase in whole pregnancy exposure to dust, motor vehicle emissions, oil combustion, and regional sulfur were 1.01 (95% CI: 0.93, 1.09), 1.01 (95% CI: 0.92, 1.10), 1.00 (95% CI: 0.89, 1.12), and 1.09 (95% CI: 0.97, 1.22) respectively.

It is unclear as to whether the statistically nonsignificant but elevated associations observed for exposure to regional sulfur (*p* = 0.130) relate to the adverse associations observed in past studies (Stieb et al. 2012) or whether this result is attributable to regional sulfur being the greatest contributor to total mass concentration in Connecticut. A possible explanation is that sulfur is a regional pollutant with little spatial variability, and thus less exposure error. Moreover, high sulfur episodes correlate with high ozone levels. It is also unclear as to why there was a decrease in odds of preterm birth associated with increases in second-trimester exposure to oil combustion and dust among Hispanic women. Possible explanations are the lack of existence of a true underlying association and the result due to random chance, that the result is confounded by negative correlation with the true underlying source, or a true underlying protective association. We conducted a post hoc analysis, with adjustment for regional sulfur exposure, and found no discernible difference with the original effect estimate. However, the value of this comparison is limited by collinearity between regional sulfur and other sources (Mostofsky et al. 2012). Because the contributions of oil combustion and dust to total mass were low, it is plausible that the unexpected results are attributable to higher uncertainty (variability) for these factors. Future studies in high exposure settings for particular sources might address this issue along with the issue of collinearity between sources.

*Assumptions and implications of the study design*. The retrospective longitudinal study design was implemented by matching pregnancies to the same woman. Unlike the case–control study design, our approach does not require a population to be defined from which the controls were obtained, and does not require adjustment of time invariant confounders on which the cases and controls were not matched (Wacholder et al. 1992). Our approach is strongly related to the case-crossover design, which has been traditionally used to examine acute exposure–events (Maclure 1991). Our analysis made the same “transience of effect” assumption as the case-crossover design (Maclure and Mittleman 2000). That is, we assumed no interference, such that exposure in an earlier pregnancy is not carried over to influence preterm birth in a subsequent pregnancy. Similarly, we assumed no residual period effect due to within-woman trend in the outcome. That is, we adjusted for parity and maternal age to account for the change in risk of preterm birth within the study period, and assumed this adjustment was sufficient. Sensitivity analyses suggested minimal influence of such unmeasured temporal factors (see Supplemental Material, Figure S7) as “order effects” (Louis et al. 1984). Although there are similarities with the case-crossover design, our study design is conceptually different. The case-crossover design centers on the case event, which implies that more than one preterm birth per subject is represented by an additional stratum, and requires the assumption that each of these events are independent. Our design centers on the woman, which means that each woman contributes equally to the effect estimate while still using all of the available information (i.e., exposure–outcome for all pregnancies) as long as the outcomes were not all the same.

*Comparisons with other studies*. The only other study that investigated preterm birth in relation to source apportioned fine particulate matter reported an OR of 1.11 (95% CI: 1.07, 1.15) per IQR increase in diesel PM_2.5_ in Los Angeles, California (Wilhelm et al. 2011). That study applied the Chemical Mass Balance model, which requires *a priori* profiles obtained from measurements of emission sources with minimal atmospheric processing. Profiles for PMF reflect aged sources after atmospheric mixing and condensation of oxidized compounds and, therefore, possibly better reflect inhaled exposure than does Chemical Mass Balance. Moreover, most epidemiologic studies lack comprehensive information on local sources, and in these situations PMF has been demonstrated more appropriate than CMB (Lee et al. 2008). Our results might differ from the California study because *a*) we investigated individual-level risk rather than its approximation from between-women comparisons more susceptible to residual socioeconomic confounding; *b*) the California study investigated a population living within 8 km of a monitoring site, who tend to have worse risk profiles than the general population (e.g., > 60% foreign born, > 70% without private health insurance, > 50% who were < 25 or > 35 years of age); or *c*) whole pregnancy exposure to PM_2.5_ (mass concentration) was higher in the California study (mean, 18.0 μg/m^3^, IQR = 2.6 μg/m^3^) than this study (mean, 12.5 μg/m^3^, IQR = 2.3 μg/m^3^).

*Potential causal pathways and causal inference*. Pathways by which air pollution might cause preterm birth might include preeclampsia (Pereira et al. 2013), metabolic disease (Brook et al. 2008), and growth restriction (Pereira et al. 2012), all risk factors for preterm birth. Although the specific mechanisms by which PM_2.5_ may lead to preterm birth are not well understood (Xu et al. 1995), elevated exposure to PM_2.5_ might promote preterm birth by increasing susceptibility to infection (Gibbs et al. 1992; Slama et al. 2008), by interfering with placental development (Dejmek et al. 2000), or through an abnormal production or early activation of cytokines favoring inflammation, which are a part of the body’s preparation for parturition (Engel et al. 2005; Keelan et al. 2003). A recent study identified a potential pathway by which PM_2.5_ absorbance was associated with preterm pre-labor rupture of membranes (Dadvand et al. 2014). Finally, it is possible that the chemical profile of PM_2.5_ per se is a less influential determinant of adverse health effects than total particulate surface area brought into contact with lung tissue (Tran et al. 2000). If this is true, correlation between surface area and mass concentration might explain the association between preterm birth and mass concentration, as has been previously reported for mortality (Maynard and Maynard 2002). If a causal effect exists, this might also explain associations reported in previous epidemiologic studies on PM_2.5_ concentration yet no detection of an association with specific sources in this study. Another possibility is that there is insufficient variability in exposures among successive pregnancies to achieve statistical power to detect an effect. This might be the case for regional sulfur, oil combustion, and dust, all of which had a mean range in whole pregnancy exposure less than that for mass concentration. However, for motor vehicle emissions, the mean range exceeded that for mass concentration, yet there was negligible evidence for an adverse association with preterm birth.

*Limitations*. Despite starting with a sample frame of > 270,000 singleton live births without congenital anomaly, we could not ascertain associations with subtypes of preterm birth (defined by phenotype or extent of early delivery) because the longitudinal nature of the study limited sample size. Based on the interquartile range of the interbirth interval distribution of women who delivered a child in the first year of the study period, we would expect to have observed a subsequent birth (if it occurred) for half to three-quarters of women using a study period of 7 years. Exclusion of women with longer interbirth intervals might have induced a degree selection bias, for example, if PM_2.5_ sources contributed to subfertility, and subsequently longer interbirth intervals and fertility treatment–associated preterm birth. Exposures did not differ between the study, reference, and source populations (see Supplemental Material, Table S1). A further limitation of this study was that exposure was assigned based on residential address recorded at delivery. Exposure would be misclassified by residential movement during pregnancy and exposures away from the home. However, sensitivity analyses provided greater confidence that estimates were robust to residential movement (see Supplemental Material, Figures S4, S5). Finally, future longitudinal studies would benefit from additional information on risk factors for preterm birth that tend to change between pregnancy (e.g., change in partner), although we expect most to be nonassociated with exposure.

## Conclusion

This was the first study to investigate the effects of PM_2.5_ sources on preterm birth and the first study to make use of longitudinal comparisons (matching on the mother) to better address individual-level confounding potentially inherent in all past studies. The greatest contributions to total mass of fine particulate matter were regional sulfur, which peaked in the warm season, and motor vehicle emissions, which peaked in the cold season. There was insufficient evidence to suggest that exposures to dust, motor vehicle emissions, oil combustion, and regional sulfur were statistically significantly associated with risk of preterm birth in Connecticut. However, insufficient evidence for an association does not imply evidence of no association. Elevated central estimates and previously observed adverse associations with mass concentration motivate the need for further research. Future studies would benefit from study settings with high exposures of a single source to address issues relating to MDL and collinearity, and longitudinal study designs such as that adopted in this study.

## Supplemental Material

(1.1 MB) PDF
