# Supplementary material for: Sources of Fine Particulate Matter and Risk of Preterm Birth in Connecticut, 2000–2006: A Longitudinal Study
Source: Environ Health Perspect. 2014 May 23;122(10):1117–22. doi: 10.1289/ehp.1307741 (PMC4181926; doi:10.1289/ehp.1307741)
Supplement: (1.1 MB) PDF [file ehp.1307741.s001.508.pdf]

Environmental Health Perspectives doi:10.1289/ehp.1307741

Supplemental Material

Sources of Fine Particulate Matter and Risk of Preterm Birth in Connecticut, 2000–2006: A Longitudinal Study

Gavin Pereira, Michelle L. Bell, Hyung Joo Lee, Petros Koutrakis, and Kathleen Belanger

Table of Contents **Page**

Table S1. Socio-demographics and characteristics of pregnancy at study entry, and whole pregnancy exposure to $PM_{2.5}$ sources, including all births for each woman in the longitudinal study population (N=23,123 women), the reference population (N=152,934 women), and the source population before exclusions (N=209,466 women) **3**

Table S2. Percentage of measurements (frequency 2-3 days) less than the Minimum Detection Limit, defined as three times the Analysis Uncertainty **5**

Table S3. Percentage of each component attributable to each source in Bridgeport, CT **6**

Table S4. Percentage of each component attributable to each source in Danbury, CT **7**

Table S5. Percentage of each component attributable to each source in Hartford, CT **8**

Table S6. Percentage of each component attributable to each source in New Haven, CT **9**

Table S7. Percentage of each component attributable to each source in Norwalk, CT **10**

Table S8. Percentage of each component attributable to each source in Waterbury, CT **11**

Figure S1. Sensitivity of associations to buffer distance **12**

Figure S2. Sensitivity of associations to smoking status **13**

Figure S3. Sensitivity of associations to the women's certainty of the first day of the last menstrual period (LMP) **14**

Figure S4. Sensitivity of associations to women moving residential location between pregnancies **15**

Figure S5. Sensitivity of associations to women moving residential location during pregnancy **16**

Figure S6. Sensitivity of associations to the exclusion of births by caesarean section **17**

Figure S7. Sensitivity of associations to additional adjustment for a GAM spline of conception date **18**

Figure S8. Sensitivity of associations to additional adjustment for area-level socioeconomic status (percent unemployed and median household income at the census tract level) and marital status (individual level) **19**

Table S1. Socio-demographics and characteristics of pregnancy at study entry, and whole pregnancy exposure to $PM_{2.5}$ sources, including all births for each woman in the longitudinal study population (N=23,123 women), the reference population (N=152,934 women), and the source population before exclusions (N=209,466 women).

Characteristic	Longitudinal study population[a] N (%)	Reference population[b] N (%)	Source population[c] N (%)
**Mother age**			
<20 years	2,434 (10.53)	14,668 (9.59)	18,184 (8.68)
20-24 years	4,539 (19.63)	28,339 (18.53)	36,264 (17.31)
25-29 years	6,723 (29.07)	38,284 (25.03)	51,449 (24.56)
30-34 years	7,215 (31.20	43,876 (28.68)	61,451 (29.33)
35-39 years	2,067 (8.94)	22,988 (15.03)	34,212 (16.33)
40+ years	145 (0.63)	4,779 (3.12)	7,906 (3.77)
**Mother education**			
<12 years	3,290 (14.23)	20,868 (13.65)	26,665 (12.73)
12 years	5,087 (22.00)	40,985 (26.80)	55,941 (26.71)
13-15 years	4,522 (19.56)	33,020 (21.59)	46,066 (21.99)
16 years	5,353 (23.15)	30,582 (20.00)	42,285 (20.19)
>16 years	4,644 (20.08)	25,786 (16.86)	36,068 (17.22)
**Marital status**			
Not married	6,982 (30.20)	52,563 (34.37)	69,974 (33.41)
Married	16,138 (69.79)	100,344 (65.61)	139,455 (66.58)
**Mother race/ethnicity**			
White (non-Hispanic)	15,469 (66.90)	94,464 (61.76)	129,915 (62.02)
Black (non-Hispanic)	2,321 (10.04)	18,178 (11.89)	25,347 (12.10)
Asian	838 (3.62)	7,229 (4.73)	9,824 (4.69)
Hispanic	3,942 (17.05)	28,867 (18.88)	38,689 (18.47)
Other	316 (1.37)	2,930 (1.92)	4,025 (1.92)
**Mother's parity**			
No children	14,741 (63.75)	78,558 (51.37)	112,233 (53.58)
1 child	5,369 (23.22)	43,507 (28.45)	56,046 (26.76)
2 children	1,962 (8.49)	20,625 (13.49)	26,849 (12.82)
≥3 children	1,051 (4.55)	10,244 (6.70)	12,900 (6.16)
**Smoking (per day)**			
None	21,802 (94.29)	142,138 (92.94)	195,072 (93.13)
1-9 cigarettes	633 (2.74)	4,968 (3.25)	6,652 (3.18)
10-20 cigarettes	666 (2.88)	5,158 (3.37)	6,859 (3.27)
>20 cigarettes	22 (0.10)	169 (0.11)	218 (0.10)
**Preterm birth**			
Term	45,622 (94.64)	143,145 (96.60)	193,869 (92.56)
Preterm	2,586 (5.36)	9,789 (6.40)	15,597 (7.45)
**$PM_{2.5}$ source exposure ($\mu g/m^3$)[d]**			
Dust	1.29 (0.77-1.88)	1.26 (0.73-1.79)	1.26 (0.74-1.79)
Motor vehicle emissions	3.52 (2.67-4.11)	3.60 (2.71-4.28)	3.62 (2.72-4.28)
Oil combustion	1.49 (1.23-1.73)	1.47 (1.17-1.74)	1.47 (1.17-1.75)
Regional sulfur	4.26 (3.62-5.28)	4.42 (3.70-5.51)	4.41 (3.69-5.51)

IQR: interquartile range, 25th percentile – 75th percentile.

[a]Longitudinal Study Population: Women that delivered singleton live born neonates without congenital anomaly by vaginal delivery and resident within 40 km of a monitoring station in the state of Connecticut who gave birth at least twice during the study period, 2000-2006. [b]Reference Population: Same as the Longitudinal Study Population without the restriction of at least two births in the study period. [c]Source Population: All women who delivered a singleton live born neonate without congenital anomaly in the state of Connecticut during the study period. [d]Whole pregnancy exposure to $PM_{2.5}$ sources, including all births for each women in the study, reference and source populations, respectively.

Table S2. Percentage of measurements (frequency 2-3 days) less than the Minimum Detection Limit, defined as three times the Analysis Uncertainty.

Component	Bridgeport	Danbury	Hartford	New Haven	Norwalk	Waterbury
Al	89	88	90	69	NS	NS
Ba	89	97	98	63	NS	NS
Br	96	97	98	96	NS	NS
Ca	52	56	65	45[a]	99	NS
Cl	81	85	84	73	99	97
Cu	80	86	93	64	NS	99
BC	29[a]	46[a]	57	11[a]	32[a]	19[a]
Fe	49[a]	77	85	32[a]	87	80
K	83	78	80	76	91	82
Mn	NS	NS	NS	97	NS	NS
Na	97	99	98	96	NS	NS
Ni	68	77	72	58	NS	NS
Pb	98	98	98	96	NS	NS
Si	70	70	76	54	NS	NS
S	18[a]	18[a]	22[a]	12[a]	19[a]	21[a]
Ti	70	71	76	56	NS	NS
V	50	62	56	37[a]	93	89
Zn	50	57	55	47[a]	95	82

[a]Components with at least half of the measurements above the MDL throughout the study period 2000-2006.

NS: Component did not meet the inclusion criteria for inclusion in the Source Apportionment.

Table S3. Percentage of each component attributable to each source in Bridgeport, CT.

Component	Sea salt	Oil combustion	Motor vehicle emissions	Dust	Regional sulfur
Al	0.07	8.28	0.00	76.91	14.74
Ba	1.49	8.03	80.25	6.18	4.06
Br	5.59	16.28	41.00	15.03	22.10
Ca	3.61	4.71	16.42	71.52	3.74
Cl	100.00	0.00	0.00	0.00	0.00
Cu	3.03	0.00	44.99	45.67	6.31
BC	1.95	1.99	84.71	6.56	4.79
Fe	2.53	1.38	35.31	57.43	3.35
K	4.80	12.61	26.62	38.19	17.79
Na	13.29	18.55	0.00	20.61	47.55
Ni	3.98	41.58	34.98	19.47	0.00
Pb	2.56	11.09	41.16	27.72	17.47
Si	0.00	8.68	0.66	82.14	8.51
S	0.20	9.15	18.98	3.74	67.93
Ti	1.94	3.24	14.63	73.32	6.88
V	3.89	86.36	2.57	0.00	7.19
Zn	1.32	9.41	58.24	25.77	5.27
$PM_{2.5}$	2.21	13.15	31.93	14.43	38.28

Table S4. Percentage of each component attributable to each source in Danbury, CT.

Component	Sea salt[a]	Oil combustion	Motor vehicle emissions	Dust	Regional sulfur
Al	7.12	3.14	4.47	65.80	19.47
Ba	6.22	0.00	51.48	38.26	4.04
Br	15.34	8.61	32.48	21.46	22.11
Ca	15.19	0.57	19.66	56.50	8.08
Cl	100.00	0.00	0.00	0.00	0.00
Cu	22.45	0.00	52.11	18.58	6.86
BC	0.00	5.16	81.58	9.30	3.96
Fe	8.06	0.02	32.83	45.90	13.19
K	35.09	0.00	21.16	25.78	17.97
Na	12.25	11.73	1.55	23.59	50.89
Ni	35.08	43.83	10.24	0.00	10.85
Pb	28.60	0.00	29.89	10.43	31.08
Si	0.00	3.09	6.43	79.29	11.19
S	1.02	6.24	19.09	4.39	69.26
Ti	7.48	1.85	12.44	59.00	19.23
V	6.49	75.63	5.07	9.24	3.57
Zn	39.98	0.00	47.28	7.99	4.75
$PM_{2.5}$	10.42	6.65	29.82	9.91	43.20

[a]Sea salt plus other non-identified sources.

Table S5. Percentage of each component attributable to each source in Hartford, CT.

Component	Sea salt	Oil combustion	Motor vehicle emissions	Dust	Regional sulfur
Al	1.38	5.84	0.00	75.74	17.04
Ba	0.29	0.00	67.66	25.65	6.40
Br	4.99	24.77	22.23	29.26	18.75
Ca	3.89	3.56	23.40	65.92	3.22
Cl	100.00	0.00	0.00	0.00	0.00
Cu	5.81	0.00	54.90	36.10	3.20
BC	0.08	4.54	91.00	0.00	4.39
Fe	2.02	1.29	44.72	46.85	5.12
K	3.72	11.70	38.74	42.38	3.47
Na	8.03	21.44	0.00	12.69	57.84
Ni	2.25	55.87	21.39	20.49	0.00
Pb	4.33	10.45	46.50	21.13	17.59
Si	0.00	10.14	0.00	86.06	3.80
S	0.00	7.33	17.06	6.21	69.39
Ti	0.10	1.49	19.37	70.06	8.97
V	0.90	91.39	1.57	0.00	6.14
Zn	1.98	7.18	70.82	20.02	0.00
$PM_{2.5}$	1.39	12.22	34.17	14.11	38.11

Table S6. Percentage of each component attributable to each source in New Haven, CT.

Component	Sea salt	Oil combustion	Motor vehicle emissions	Dust	Regional sulfur
Al	1.62	0.00	7.50	70.51	20.37
Ba	0.45	8.93	10.66	70.42	9.54
Br	6.12	7.34	45.17	0.00	41.37
Ca	4.01	0.86	20.03	64.94	10.16
Cl	99.61	0.38	0.01	0.00	0.00
Cu	1.61	5.91	23.64	46.71	22.12
BC	1.38	4.23	67.93	18.40	8.07
Fe	1.82	5.61	14.43	67.33	10.81
K	4.81	2.09	35.22	29.07	28.81
Mn	3.74	1.34	28.83	53.52	12.57
Na	17.00	3.87	0.00	22.04	57.09
Ni	3.97	68.52	10.49	11.33	5.68
Pb	3.13	2.14	56.98	9.80	27.95
Si	1.93	0.00	5.52	81.48	11.07
S	0.46	6.13	9.13	9.49	74.79
Ti	1.32	1.84	4.41	72.33	20.10
V	0.59	87.38	3.39	5.08	3.56
Zn	3.54	14.57	57.86	14.21	9.82
$PM_{2.5}$	3.60	2.98	30.49	9.13	53.80

Table S7. Percentage of each component attributable to each source in Norwalk, CT.

Component	Oil combustion	Motor vehicle emissions	Dust	Regional sulfur
Ca	7.94	24.17	46.55	21.33
Cl	20.89	32.62	46.49	0.00
BC	13.48	52.10	27.33	7.09
Fe	10.01	34.57	38.96	16.46
K	8.40	23.70	45.80	22.10
S	13.79	23.74	3.25	59.22
V	67.55	10.19	7.62	14.64
Zn	15.19	41.41	35.36	8.04
$PM_{2.5}$	14.43	29.44	17.64	38.50

Table S8. Percentage of each component attributable to each source in Waterbury, CT.

Component	Oil combustion	Motor vehicle emissions	Dust	Regional sulfur
Cl	34.55	0.32	65.13	0.00
Cu	14.26	51.91	28.40	5.43
BC	14.04	56.78	20.28	8.90
Fe	13.29	43.16	30.63	12.93
K	29.12	28.62	30.52	11.74
S	15.56	20.06	2.60	61.78
V	76.43	8.34	4.54	10.69
Zn	7.99	22.36	61.33	8.32
$PM_{2.5}$	20.72	29.18	14.25	35.85

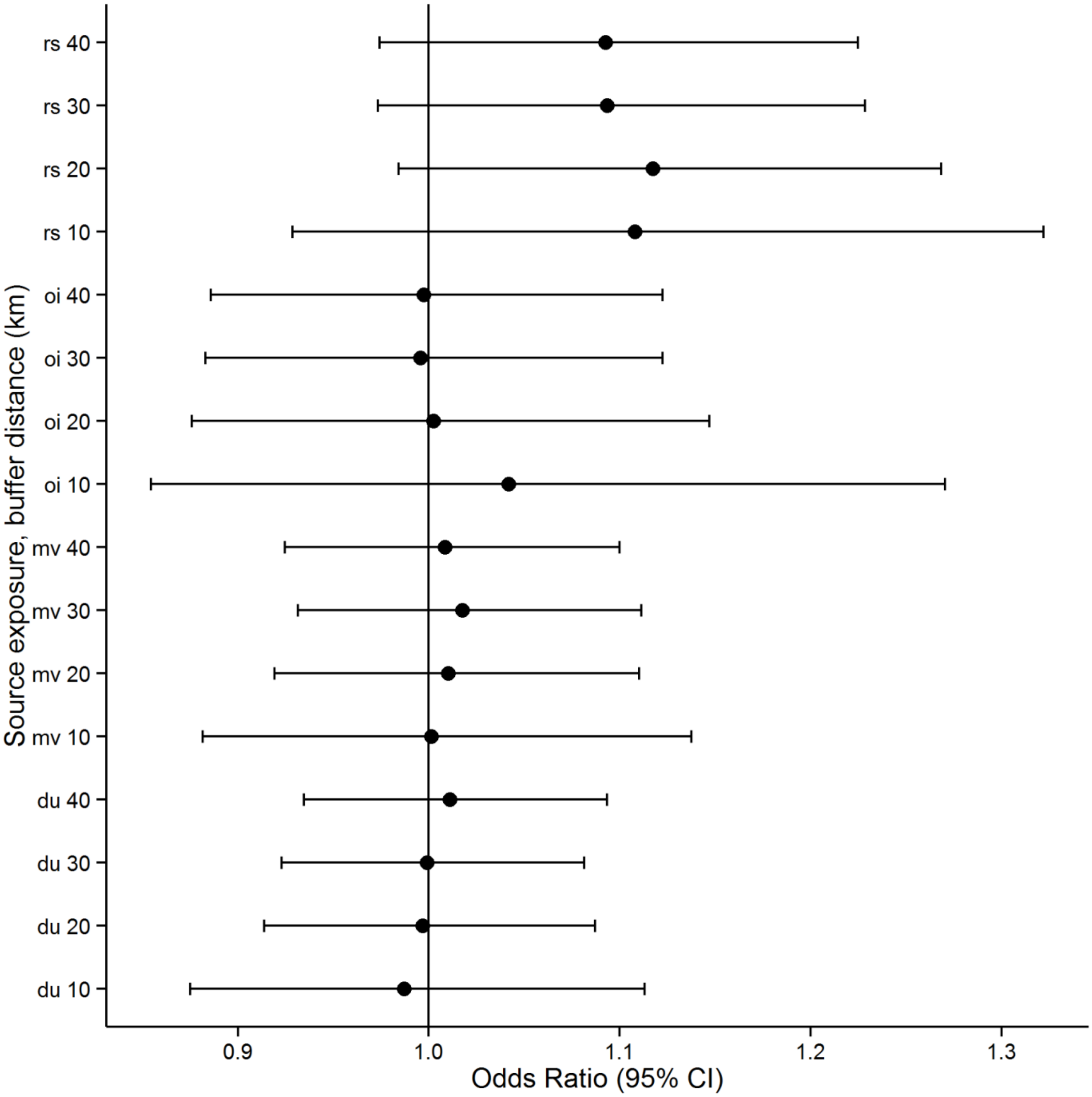

Figure S1. Sensitivity of associations to buffer distance. Adjusted preterm birth odds ratios and 95% confidence intervals per interquartile range increase in whole pregnancy exposure to regional sulfur (rs), oil combustion (oi), motor vehicle emissions (mv), and dust (du) including women within buffer distances of 40 km (original analyses), and 30 km, 20 km and 10 km.

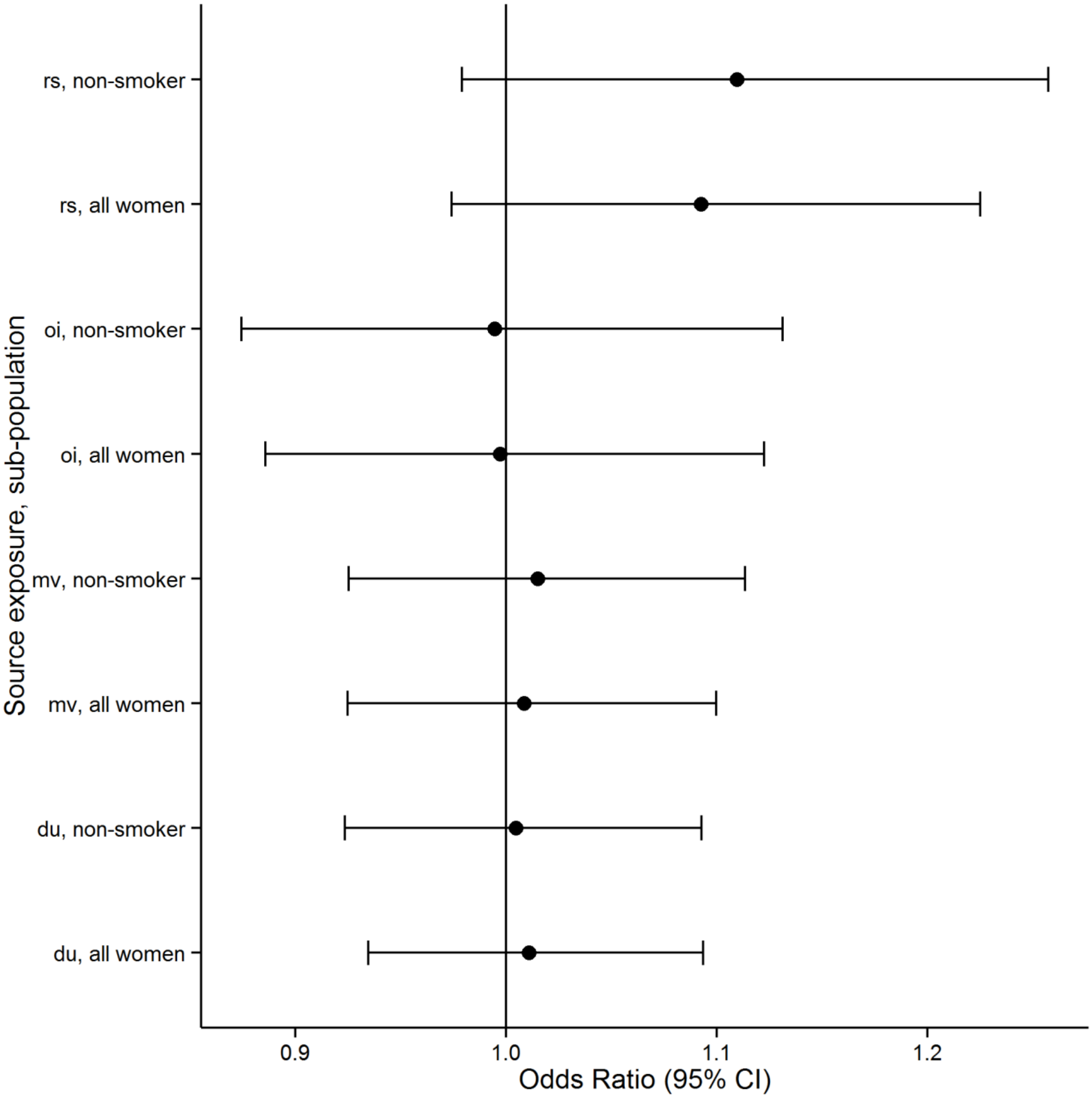

Figure S2. Sensitivity of associations to smoking status. Adjusted preterm birth odds ratios and 95% confidence intervals per interquartile range increase in whole pregnancy exposure to regional sulfur (rs), oil combustion (oi), motor vehicle emissions (mv), and dust (du) for all women irrespective of smoking status (original analyses), and non-smokers (<1 cigarette per day).

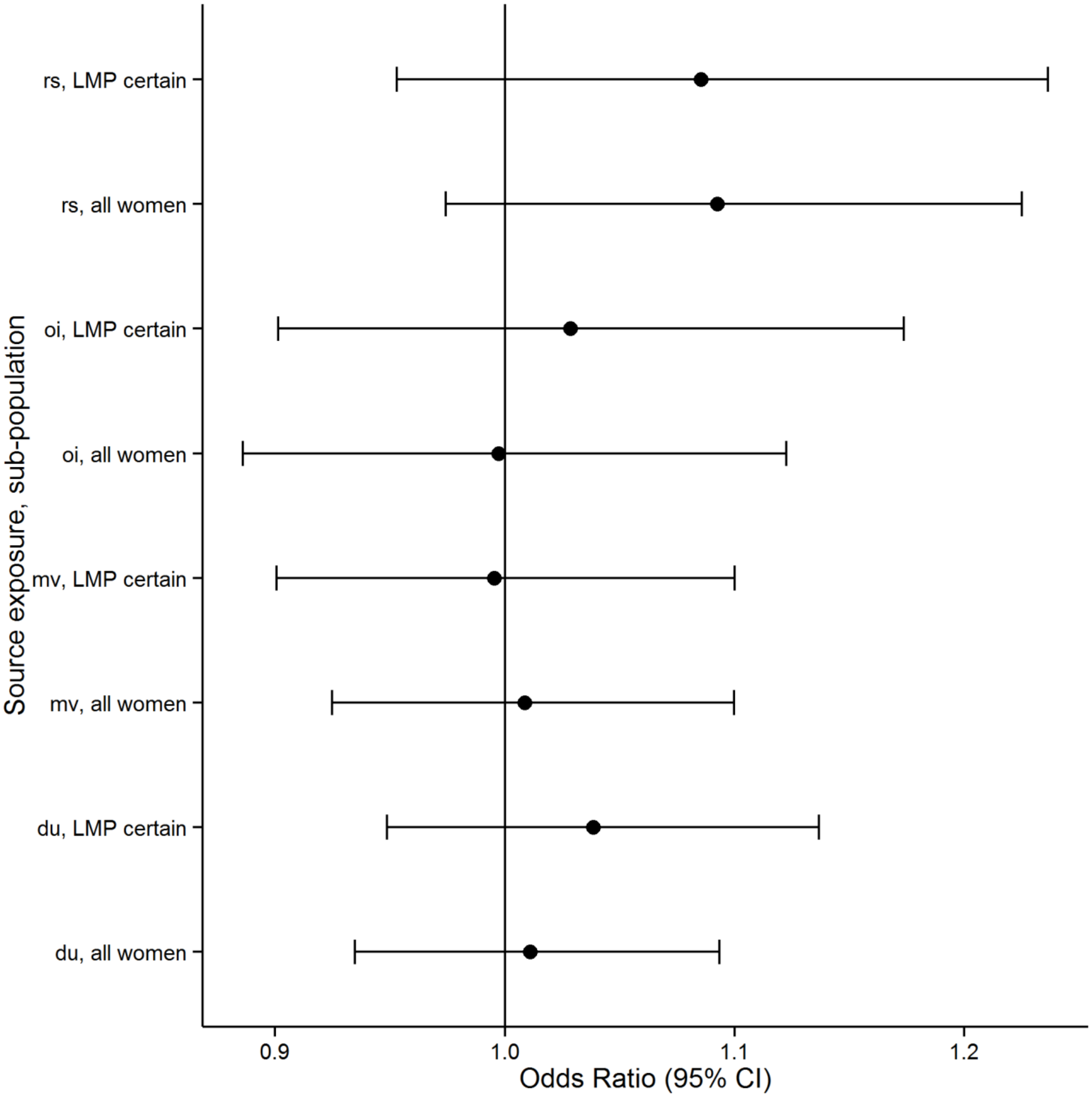

Figure S3. Sensitivity of associations to the women's certainty of the first day of the last menstrual period (LMP). Adjusted preterm birth odds ratios and 95% confidence intervals per interquartile range increase in whole pregnancy exposure to regional sulfur (rs), oil combustion (oi), motor vehicle emissions (mv), and dust (du) for all women irrespective of LMP certainty (original analyses), and women certain about their LMP date.

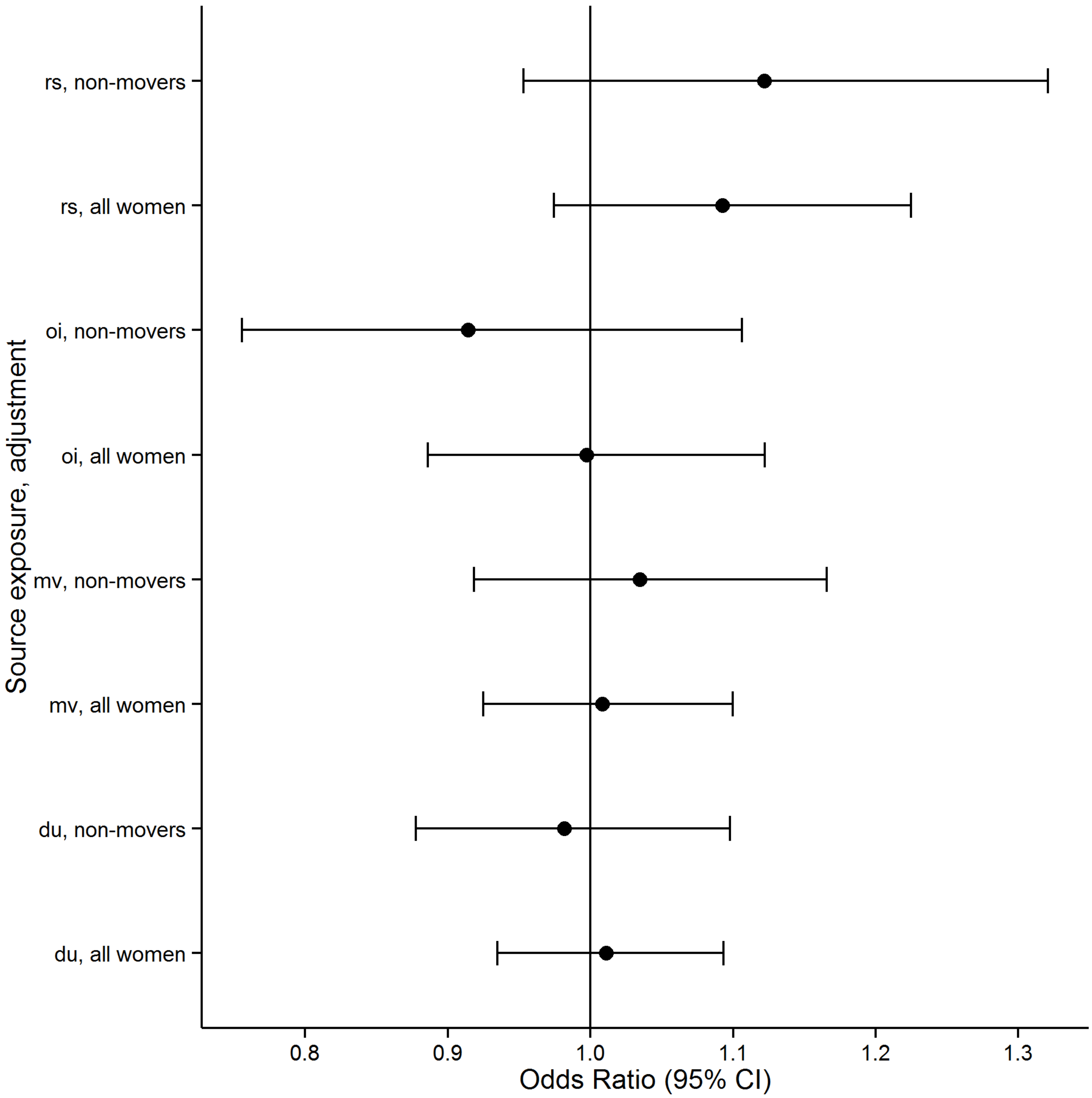

Figure S4. Sensitivity of associations to women moving residential location between pregnancies. Adjusted preterm birth odds ratios and 95% confidence intervals per interquartile range increase in whole pregnancy exposure to regional sulfur (rs), oil combustion (oi), motor vehicle emissions (mv), and dust (du) for all women irrespective of moving home (all women), and women whose address recorded at delivery did not differ between births (non-movers).

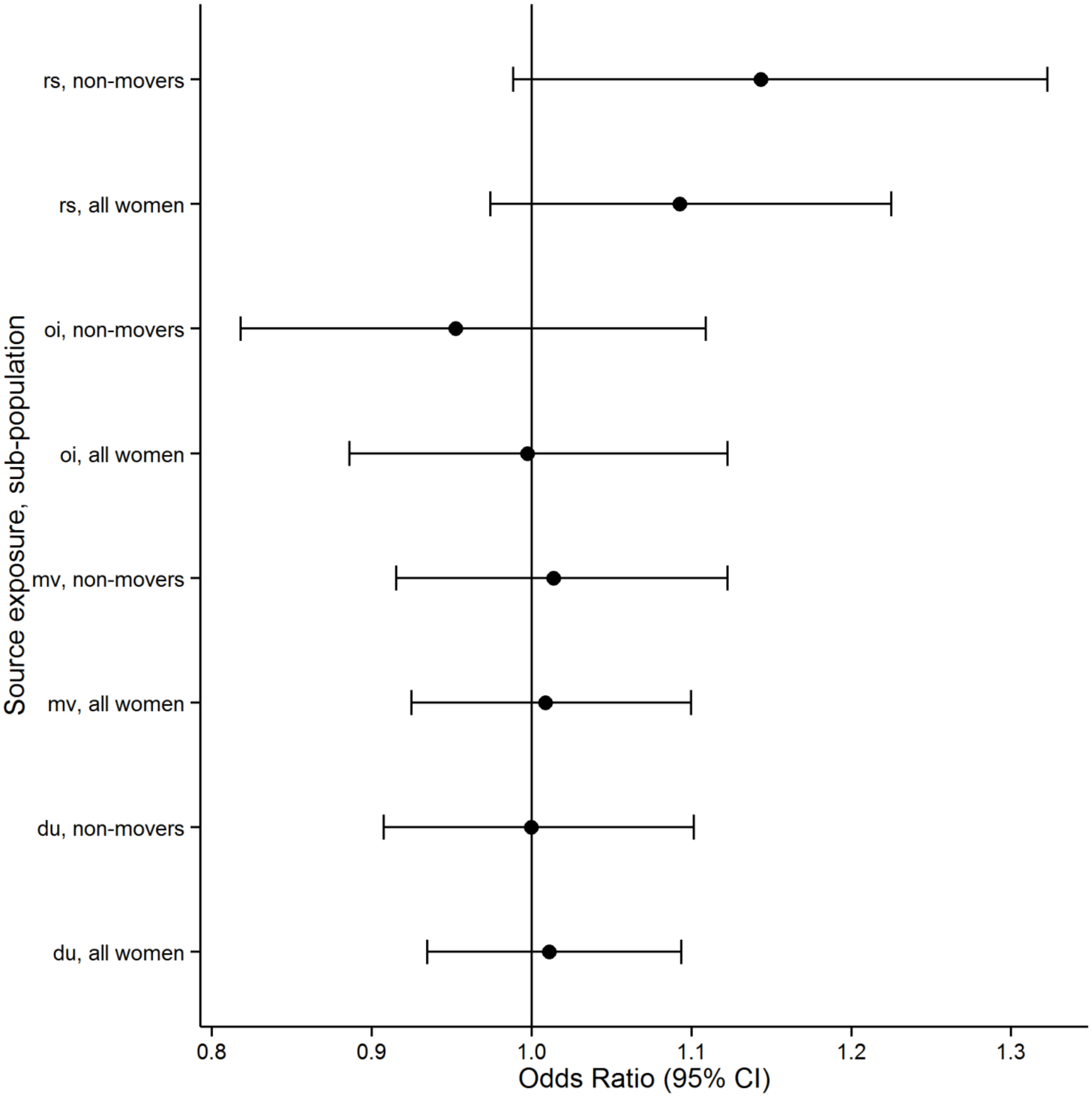

Figure S5. Sensitivity of associations to women moving residential location during pregnancy. Adjusted preterm birth odds ratios and 95% confidence intervals per interquartile range increase in whole pregnancy exposure to regional sulfur (rs), oil combustion (oi), motor vehicle emissions (mv), and dust (du) for all women irrespective of moving home (all women), and women who remained at their address for the duration of their pregnancy (non-movers).

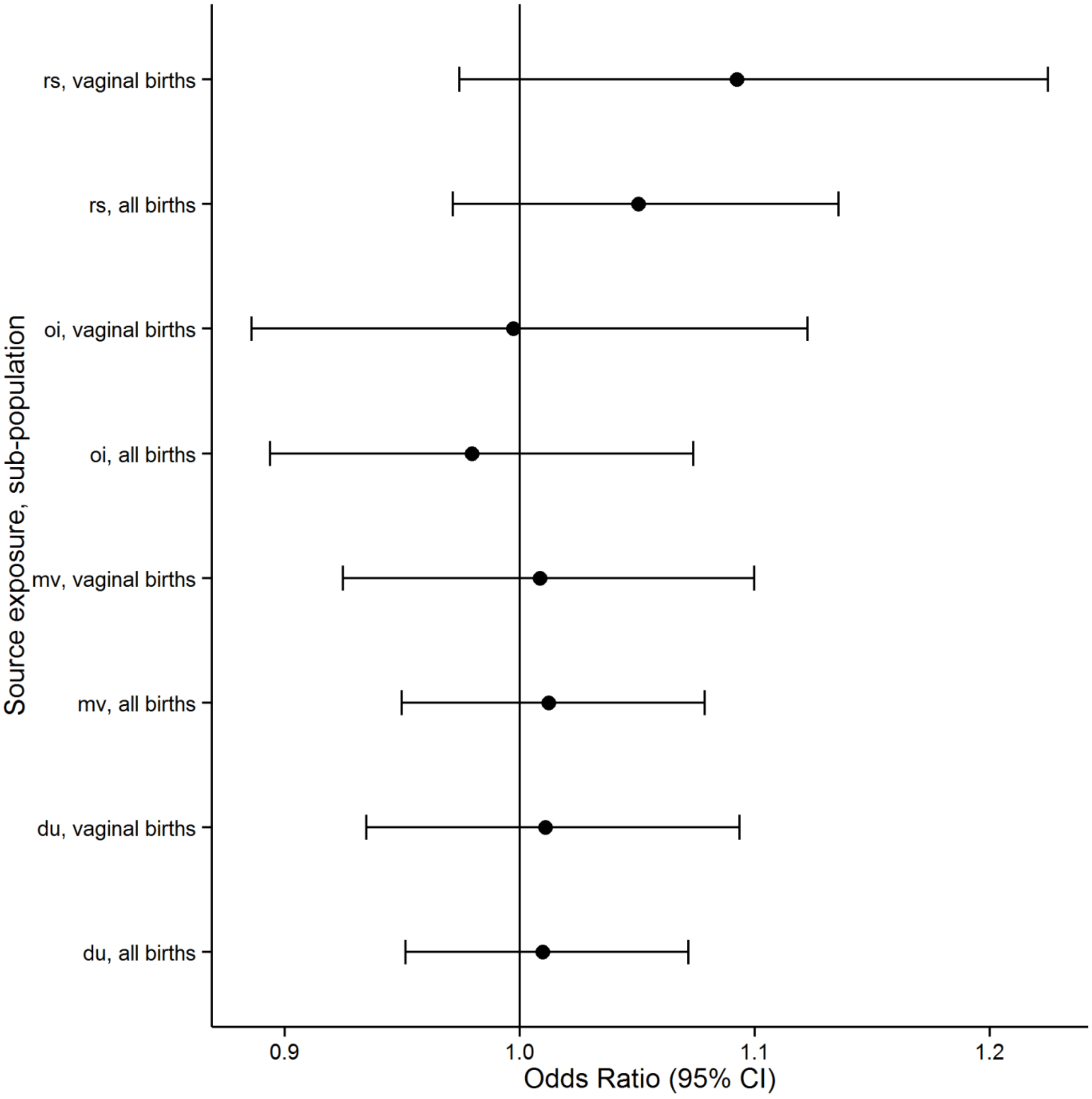

Figure S6. Sensitivity of associations to the exclusion of births by caesarean section. Adjusted preterm birth odds ratios and 95% confidence intervals per interquartile range increase in whole pregnancy exposure to regional sulfur (rs), oil combustion (oi), motor vehicle emissions (mv), and dust (du) for vaginal births (original analyses), and all births (vaginal births and caesarean sections).

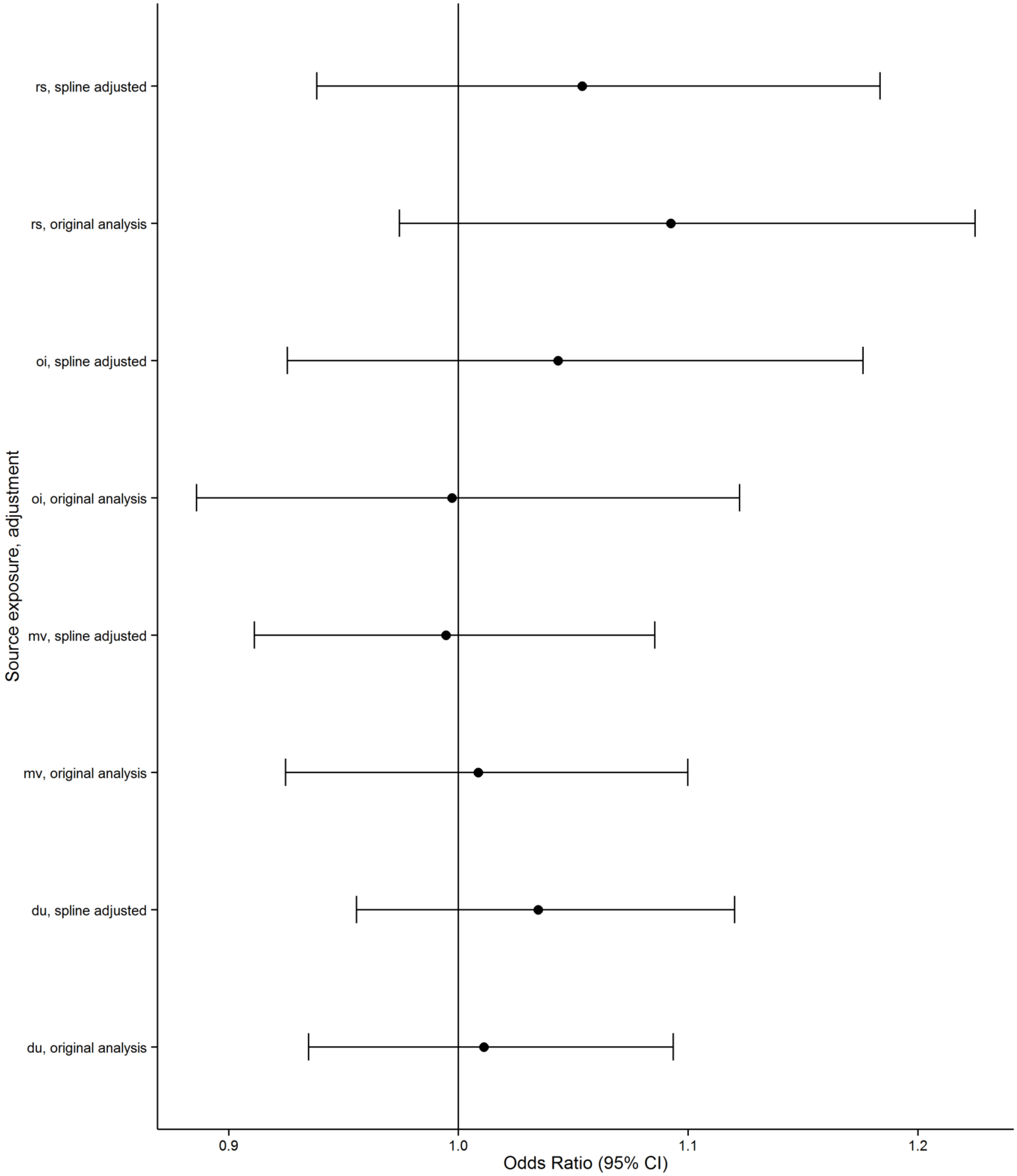

Figure S7. Sensitivity of associations to additional adjustment for a GAM spline of conception date. The smoothing parameter was selected by generalized cross-validation. Adjusted preterm birth odds ratios and 95% confidence intervals per interquartile range increase in whole pregnancy exposure to regional sulfur (rs), oil combustion (oi), motor vehicle emissions (mv), and dust (du) for the original analyses, and with additional adjustment for a GAM spline of conception date.

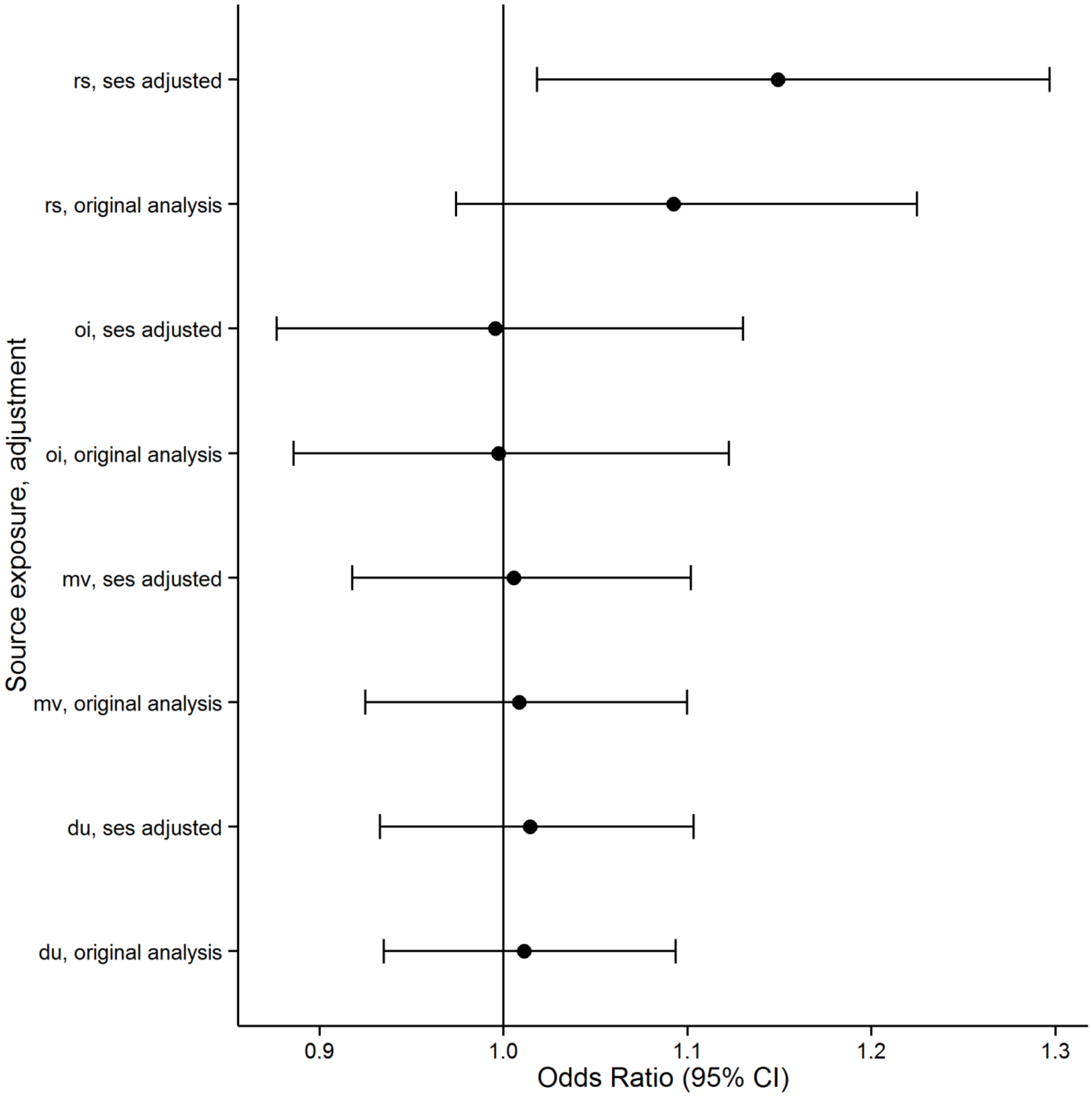

Figure S8. Sensitivity of associations to additional adjustment for area-level socioeconomic status (percent unemployed and median household income at the census tract level) and marital status (individual level). Adjusted preterm birth odds ratios and 95% confidence intervals per interquartile range increase in whole pregnancy exposure to regional sulfur (rs), oil combustion (oi), motor vehicle emissions (mv), and dust (du) with for the original analyses, and with adjustment for additional SES variables.
